# Insights into the inhibitory mechanisms of NADH on the αγ heterodimer of human NAD-dependent isocitrate dehydrogenase

**DOI:** 10.1038/s41598-018-21584-7

**Published:** 2018-02-16

**Authors:** Yabing Liu, Lejia Hu, Tengfei Ma, Jun Yang, Jianping Ding

**Affiliations:** 1 School of Life Sciences, Shanghai University, 333 Nanchen Road, Shanghai, 200444 China; 2 State Key Laboratory of Molecular Biology, National Center for Protein Science Shanghai, Shanghai Science Research Center, CAS Center for Excellence in Molecular Cell Science, Institute of Biochemistry and Cell Biology, Shanghai Institutes for Biological Sciences, Chinese Academy of Sciences, 320 Yue-Yang Road, Shanghai, 200031 China; 3 School of Life Science and Technology, ShanghaiTech University, 393 Hua-Xia Zhong Road, Shanghai, 201210 China

## Abstract

Human NAD-dependent isocitrate dehydrogenase (NAD-IDH) catalyzes the oxidative decarboxylation of isocitrate in the citric acid cycle. In the α_2_βγ heterotetramer of NAD-IDH, the γ subunit plays the regulatory role and the β subunit the structural role. Previous biochemical data have shown that mammalian NAD-IDHs can be inhibited by NADH; however, the molecular mechanism is unclear. In this work, we show that the αβ, αγ and α_2_βγ enzymes of human NAD-IDH can be inhibited by NADH, and further determine the crystal structure of the αγ heterodimer bound with an Mg^2+^ and an NADH at the active site and an NADH at the allosteric site, which resembles that of the inactive α^Mg^γ heterodimer. The NADH at the active site occupies the binding site for NAD^+^ and prevents the binding of the cofactor. The NADH at the allosteric site occupies the binding sites for ADP and citrate and blocks the binding of the activators. The biochemical data confirm that the NADH binding competes with the binding of NAD^+^ and the binding of citrate and ADP, and the two effects together contribute to the NADH inhibition on the activity. These findings provide insights into the inhibitory mechanisms of the αγ heterodimer by NADH.

## Introduction

Isocitrate dehydrogenases (IDHs) are the enzymes that catalyze the oxidative decarboxylation of isocitrate (ICT) into α-ketoglutarate (α-KG) and CO_2_ while converting the coenzyme NAD^+^ or NADP^+^ into NADH or NADPH. In mammals, the mitochondria localized NAD-dependent IDHs (NAD-IDHs) are deemed to exert the catalytic role in the citric acid cycle. Mammalian NAD-IDH functions as a heterotetramer consisting of two α subunits (37 kDa), one β subunit (39 kDa), and one γ subunit (39 kDa). The α and β subunits share about 40% sequence identity, the α and γ subunits about 42% sequence identity, and the β and γ subunits about 52% sequence identity^1,2^. The α and β subunits form a heterodimer (αβ) and the α and γ subunits form another (αγ), and the two heterodimers are assembled into the heterotetramer (α_2_βγ), which can be further assembled into a heterooctamer^3,4^.

Previous biochemical studies have shown that the enzymatic activity of mammalian NAD-IDH could be positively regulated by citrate (CIT) and ADP; and in the α_2_βγ heterotetramer, the α subunit exerts the catalytic role, and the β and γ subunits play the regulatory roles^5–11^. In our previous biochemical studies, we demonstrated that the α_2_βγ heterotetramer and the αγ heterodimer of human NAD-IDH could be activated by CIT and ADP, whereas the αβ heterodimer cannot; and the β subunit plays the structural role in the assembly and the γ subunit the regulatory role in the function of the heterotetramer^12^. Due to the lack of structural information about the α_2_βγ heterotetramer, the molecular basis for the assembly of the α_2_βγ heterotetramer and the molecular mechanisms of the allosteric regulation of the α_2_βγ heterotetramer are still elusive. Although there is no evidence so far showing that the αβ and αγ heterodimers can exist alone in the cells and play any physiological roles, we have carried out detailed structural and functional studies of the αβ and αγ heterodimers, hoping that these studies could provide insights into the structure, function and allosteric regulation of the α_2_βγ heterotetramer. Our previous structural studies of the αγ heterodimer reveal that the binding of CIT and ADP to the allosteric site in the γ subunit causes conformational changes of the allosteric site, the heterodimer interface, and the active site in the α subunit in a concerted way, leading to decrease of the *S*_0.5,ICT_ and thus activation of the enzyme^13^. The functional roles of some key residues participating in the ligand binding and structural changes in the αγ heterodimer and the α_2_βγ heterotetramer have been confirmed by mutagenesis and enzymatic studies^12,13^. The biochemical and structural data together provide insights into the molecular mechanism of the activation of the αγ heterodimer by CIT and ADP, and additionally indicate that the structural information derived from the αγ heterodimer might be applicable to the α_2_βγ heterotetramer.

Previous biochemical studies have also shown that the enzymatic activity of mammalian NAD-IDH could be inhibited by NADH^5,14^; however, the inhibition mechanism is unclear. In this work, we examined the inhibition of the αβ and αγ heterodimers and the α_2_βγ heterotetramer of human NAD-IDH by NADH and found that all these enzymes can be substantially inhibited by NADH. To understand the molecular mechanism of the NADH inhibition, we solved the crystal structure of the αγ heterodimer bound with an Mg^2+^ and an NADH at the active site and an NADH at the allosteric site. The overall structure of the α^Mg+NADH^γ^NADH^ heterodimer resembles that of the α^Mg^γ heterodimer, representing the inactive state of the enzyme. Structure analysis shows that the bound NADH at the active site occupies the binding site for NAD^+^, thus preventing the binding of the cofactor; and the bound NADH at the allosteric site occupies a large portion of the binding sites for ADP and CIT, thus blocking the binding of the activators. The kinetic data confirm that the NADH binding competes with the binding of NAD^+^ to the active site and the binding of CIT and ADP to the allosteric site, and the two effects together contribute to the inhibition of NADH on the αγ heterodimer. These findings provide insights into the inhibitory mechanism of the αγ heterodimer by NADH.

## Results and Discussion

### NADH inhibits the activities of the αγ, αβ and α_2_βγ enzymes of human NAD-IDH

**Figure 1:**
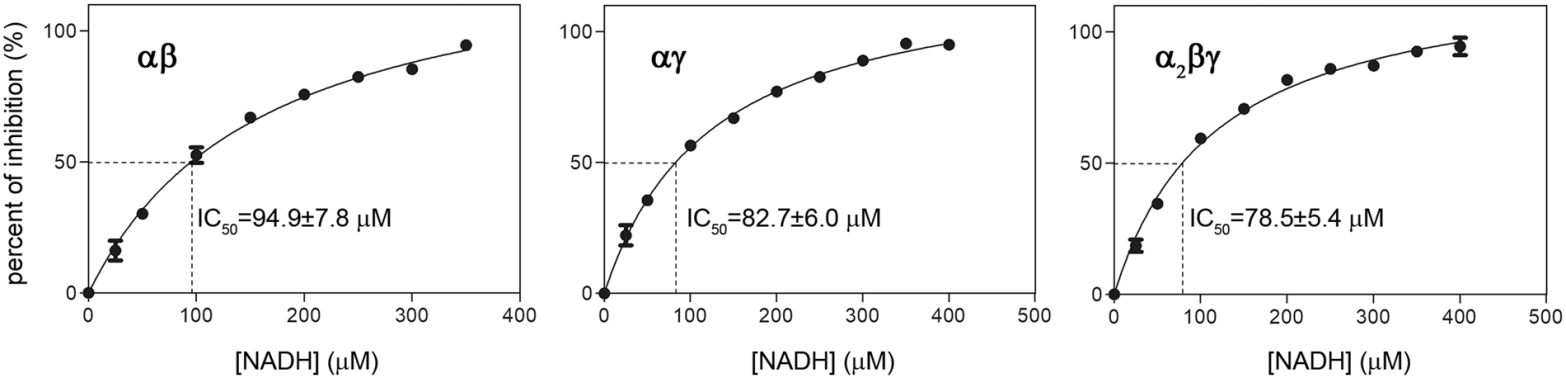
Inhibitory effects of NADH on the αγ, αβ and α_2_βγ enzymes of human NAD-IDH. The enzymatic activities of the αγ and α_2_βγ enzymes in the presence of NADH were measured at the standard conditions with varied concentrations of NADH as described in “Methods”. The activity of the αβ enzyme was determined at the same conditions except for a higher concentration of MnCl_2_ (50 mM). The inhibitory effect of NADH on the activity of the enzyme is presented as percent of inhibition (I) as a function of NADH concentration. I = (*V*_0_−*V*)/*V*_0_ * 100, where *V*_0_ and *V* are the initial velocities in the absence and presence of NADH, respectively.

The previous biochemical studies have shown that the enzymatic activity of NAD-IDH purified from bovine heart can be inhibited by NADH^5,14^. In this study, we first examined the inhibitory effect of NADH on the activities of the αβ and αγ heterodimers and the α_2_βγ heterotetramer of human NAD-IDH (Fig. 1). At the standard conditions (33 mM Tris-acetate, pH 7.4, 2 mM MnCl_2_, 20 mM _DL_-ICT, and 1 mM NAD^+^), the α_2_βγ heterotetramer and the αγ heterodimer exhibit a specific activity of 24.0 ± 0.5 and 6.75 ± 0.07 μmol/min/mg in the absence of NADH, and NADH can substantially inhibit the activity of both enzymes with an IC_50,NADH_ of 78.5 ± 5.4 μM and 82.7 ± 6.0 μM, respectively. As the αβ heterodimer shows a very low activity at the standard conditions (1.15 ± 0.26 μmol/min/mg) and has a much higher *S*_0.5,Mn_ (5.3 mM) than the α_2_βγ and αγ enzymes (*S*_0.5,Mn_ of 0.060 mM and 0.095 mM, respectively)^12^, we measured the inhibitory effect of NADH on the αβ heterodimer at a higher Mn^2+^ concentration (50 mM). At that conditions, the αβ heterodimer exhibits an activity of 7.22 ± 0.31 μmol/min/mg in the absence of NADH, which can also be inhibited by NADH with an IC_50,NADH_ of 94.9 ± 7.8 μM. These results indicate that NADH can bind to and inhibit the activities of the αβ, αγ and α_2_βγ enzymes of human NAD-IDH.

**Figure 2:**
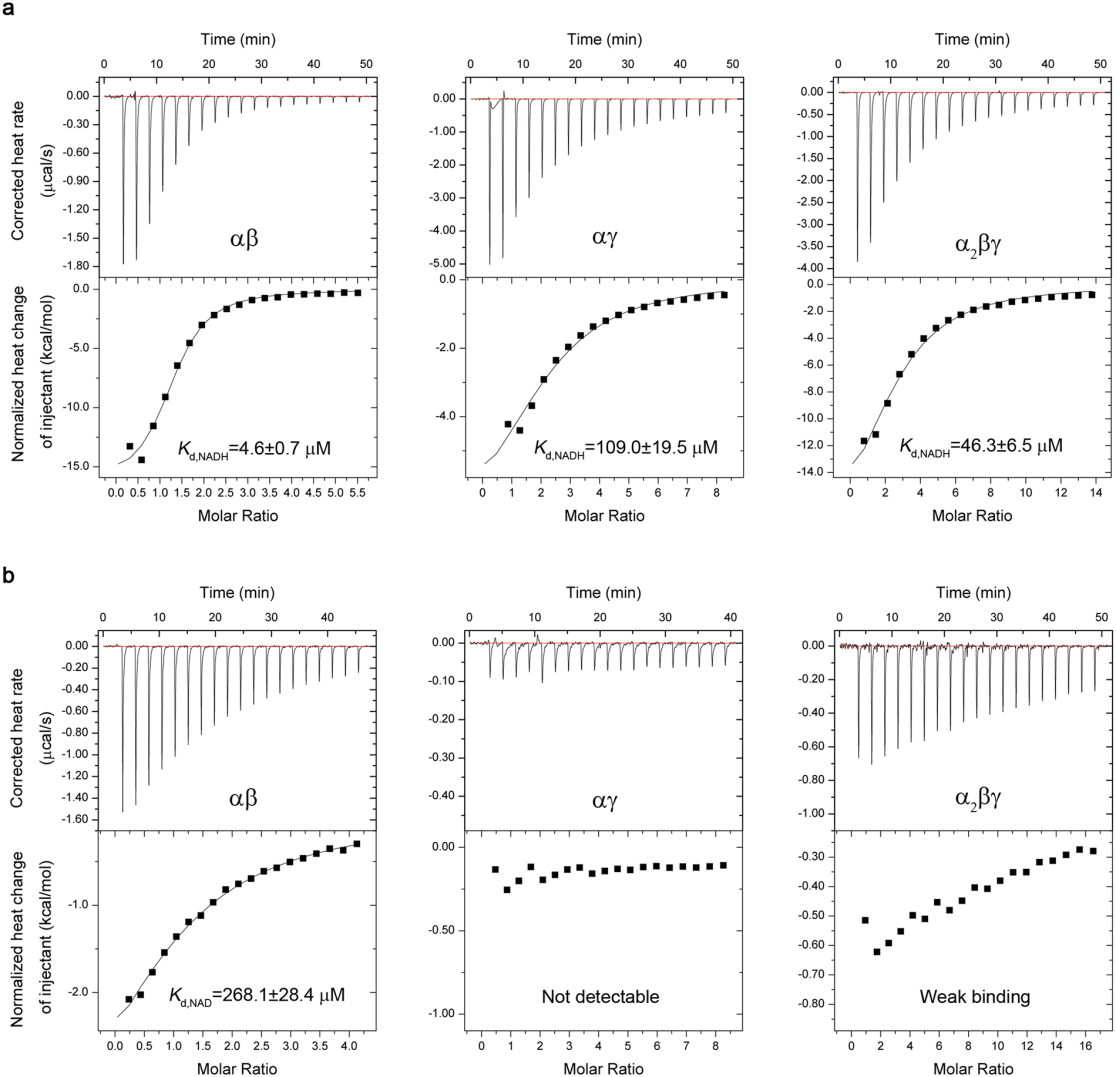
Binding of NADH (**a**) and NAD^+^ (**b**) with the αγ, αβ and α_2_βγ enzymes of human NAD-IDH measured by ITC at 20 °C. The concentrations of titrand (protein) and titrant (NADH or NAD^**+**^) in the measurements are listed in Table 1. The corrected heat change was obtained by subtracting the heat of dilution from the measured heat change, and the normalized corrected heat change of injectant was plotted against the molar ratio of titrant vs. titrand. The values of thermodynamic parameters were derived from the titration curve by fitting the experimental data using a nonlinear least-squares method with the single set of binding sites model and are summarized in Table 1. The dissociation constant *K*_d_ values are shown in the figure.

We then analyzed the binding abilities of NADH with the αβ and αγ heterodimers and the α_2_βγ heterotetramer using the isothermal titration calorimetry (ITC) method. The ITC results show that all three enzymes can bind NADH, and the αβ heterodimer has the highest binding affinity (*K*_d_ = 4.6 ± 0.7 μM) followed by the α_2_βγ heterotetramer (*K*_d_ = 46.3 ± 6.5 μM) and then the αγ heterodimer (*K*_d_ = 109.0 ± 19.5 μM) (Fig. 2a and Table 1). Furthermore, we analyzed the binding of NAD^+^ with the three enzymes in the absence of the substrate ICT and the activators CIT and ADP. The ITC results show that only the αβ heterodimer has a measurable binding for NAD^+^ (*K*_d_ = 268.1 ± 28.4 μM), whereas the α_2_βγ heterotetramer has a very weak binding and the αγ heterodimer has no detectable binding (Fig. 2b and Table 1). These results indicate that all three enzymes have much tighter binding for NADH than for NAD^+^, which is consistent with the enzymatic data showing that 100 μM NADH could inhibit >50% activities of the enzymes in the presence of 2 mM NAD^+^ (Fig. 1).

**Table 1:** Thermodynamic parameters of NADH and NAD^+^ binding with the αβ, αγ and α_2_βγ enzymes of human NAD-IDH analyzed by ITC at 20 °C.

Protein	*K*_*d*_ *(μ* *M)* ^a^	*ΔH (kcal/mol)*	*TΔS*	*n*-value
For NADH				
αβ (30 μM/0.8 mM)^b^	4.6 ± 0.7	−16.6 ± 0.7	−9.03	1.26 ± 0.04
αγ (100 μM/4 mM)	109.0 ± 19.5	−8.1 ± 1.3	−2.62	2.19 ± 0.27
α_2_βγ (30 μM/2 mM)	46.3 ± 6.5	−21.8 ± 2.7	−14.8	2.53 ± 0.25
For NAD^**+**^				
αβ (200 μM/4 mM)	268.1 ± 28.4	−5.0 ± 0.7	−0.2	1.14 ± 0.11
αγ (100 μM/4 mM)	ND	ND	ND	ND
α_2_βγ (100 μM/8 mM)	WB	WB	WB	WB

^a^Abbreviations: *K*_*d*_, dissociation constant; ND, not detectable; WB, weak binding.

^b^Numbers in parentheses refer to the concentrations of titrand (protein)/titrant (NADH or NAD^**+**^) in the measurements.

In addition, the thermodynamic data reveal the n-value of the αβ and αγ heterodimers and the α_2_βγ heterotetramer for NADH as 1.26 ± 0.04, 2.19 ± 0.27, and 2.53 ± 0.25, respectively, and the n-value of the αβ heterodimer for NAD^+^ as 1.14 ± 0.11, indicating that the αβ heterodimer has one binding site for NADH or NAD^+^, whereas the αγ heterodimer and the α_2_βγ heterotetramer have at least two binding sites for NADH. These results are in agreement with our structural data demonstrating that the αγ heterodimer contains two NADH-binding sites (see discussion later). As the αβ heterodimer contains only one NADH-binding site, it is very likely that NADH binds to the active site in the α subunit and hence prevents the binding of the cofactor and inhibits the activity of the enzyme. Nevertheless, it is unclear why the αβ heterodimer exhibits stronger binding affinities for both NADH and NAD^+^ than the αγ heterodimer and the α_2_βγ heterotetramer. One possibility is that the ability of the γ subunit and/or the inability of the β subunit to bind NADH might have different effects on the binding of NADH or NAD^+^ at the active site in the α subunit. Further structural and functional studies of the αβ heterodimer and the α_2_βγ heterotetramer will resolve this issue and uncover the inhibitory mechanisms of the αβ heterodimer and the α_2_βγ heterotetramer by NADH.

### Overall structure of the α^Mg+NADH^γ^NADH^ heterodimer

**Figure 3:**
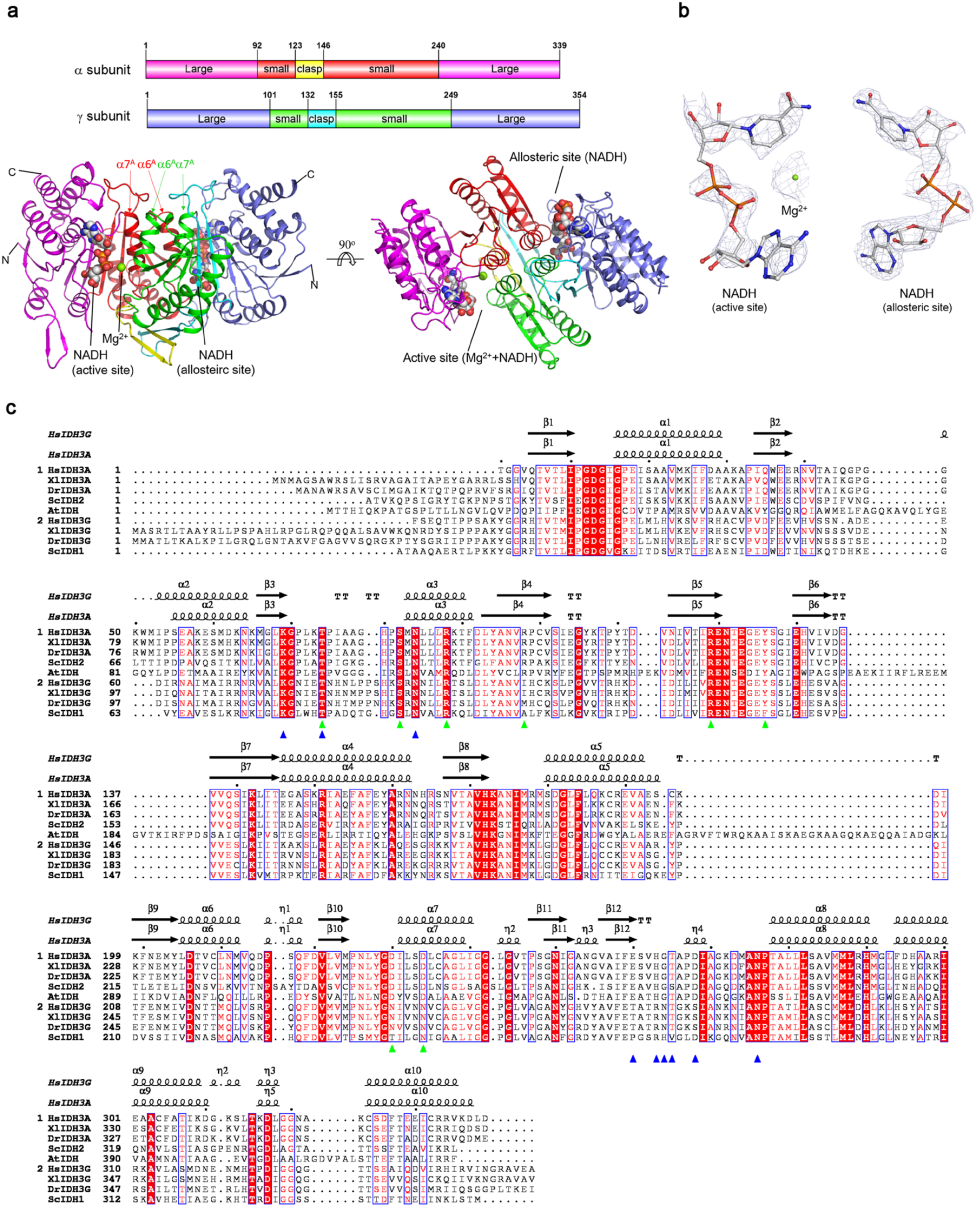
Structure of the α^Mg+NADH^γ^NADH^ heterodimer of human NAD-IDH. (**a**) Overall structure of the α^Mg+NADH^γ^NADH^ heterodimer in two different views. Left: view in perpendicular to the pseudo 2-fold axis of the αγ heterodimer. Right: view along the pseudo 2-fold axis of the αγ heterodimer. The color-coding schemes of individual domains of the α and γ subunits are shown above. The bound NADHs are shown in space-filling models, and the bound Mg^2+^ ion as a green sphere. (**b**) Representative simulated annealing composite omit maps (contoured at 1.0σ level) of the bound NADHs in the α^Mg+NADH^γ^NADH^ structure. Left: Mg^2+^ and NADH at the active site. Right: NADH at the allosteric site. The NADH is shown in ball-and-stick model and the Mg^2+^ with a green sphere, respectively. (**c**) Structure-based sequence alignment of the α and γ subunits of human NAD-IDH with several representative NAD-IDHs. *Homo sapien* NAD-IDH: HsIDH3; *S. cerevesiae* NAD-IDH: ScIDH; *Xenopus laevis* NAD-IDH: XlIDH3; *Danio rerio* NAD-IDH: DrIDH3; *Acidithiobacillus thiooxidans* NAD-IDH: AtIDH. The secondary structures of the α and γ subunits in the α^Mg+NADH^γ^NADH^ structure are placed on the top of the alignment. Invariant residues are highlighted by shaded red boxes and conserved residues by open blue boxes. The residues corresponding to those composing the ICT-binding site and the NAD-binding site in the αγ heterodimer of human NAD-IDH are highlighted with green and blue triangles, respectively.

To understand the inhibitory mechanism of the αγ heterodimer by NADH, we solved the crystal structure of the αγ heterodimer bound with an Mg^2+^ and an NADH at the active site and an NADH at the allosteric site (α^Mg+NADH^γ^NADH^) at 2.4 Å resolution using the molecular replacement method (Table 2) (Fig. 3a). The structure belongs to space group *P*3_1_21 containing one αγ heterodimer per asymmetric unit. The majority of the residues in both α and γ subunits are well defined except for a few N-terminal and C-terminal residues due to poor electron density. There is evident electron density for a metal ion and an NADH (or NAD^+^) at the active site and an NADH (or NAD^+^) at the allosteric site (Fig. 3b). The bound metal ion was interpreted as an Mg^2+^ with a reasonable B-factor as there was 50 mM Mg^2+^ in the crystallization solution. The bound ligands were interpreted as NADH because there is only NADH but no oxidizing agent in the crystallization solution and the NADH could not be oxidized into NAD^+^ in the crystallization. The NADH at the active site has a higher B-factor than the NADH at the allosteric site and the protein, indicating a lower occupancy or higher flexibility. This may suggest that the active site has a relatively lower affinity for NADH than the allosteric site.

**Table 2:** Statistics of X-ray diffraction data and structure refinement.

	α^Mg+NADH^γ^NADH^
**Data collection**	
Wavelength (Å)	0.9785
Space group	*P*3_1_21
*a*, *b* (Å)	118.0
*c* (Å)	142.1
Resolution (Å)	50.0–2.40 (2.49–2.40)^a^
Observed reflections	449,533
Unique reflections (I/σ(I) > 0)	45,428
Average redundancy	9.9 (8.9)
Average I/σ(I)	22.6 (2.8)
Completeness (%)	100.0 (100.0)
R_merge_ (%)^b^	12.1 (65.3)
**Refinement and structure model**	
No. of reflections (*Fo* > 0σ(*Fo*))	45,386
Working set	43,063
Test set	2,323
R factor/R_free_ factor (%)^c^	21.1/23.6
Total protein atoms	5,048
Total ligand atoms	89
Total solvent atoms	94
Wilson B factor (Å^2^)	54.3
Average B factor (Å^2^)	64.5
Protein	63.9
Mg (active site)	52.6
NADH (active/allosteric site)	129.3/70.8
Water	54.3
RMS deviations	
Bond lengths (Å)	0.008
Bond angles (°)	1.3
Ramachandran plot (%)	
Most favored	96.5
Allowed	3.5

^a^Numbers in parentheses refer to the highest resolution shell.

^b^R_*merge*_ = $${\sum }_{hkl}{\sum }_{i}|{I}_{i}{(hkl)}_{i}-\langle I(hkl)\rangle |/{\sum }_{hkl}{\sum }_{i}{I}_{i}(hkl).$$

^c^R factor = ∑||F_o_| − |F_c_||/∑|F_o_|.

The overall structure of the α^Mg+NADH^γ^NADH^ heterodimer is very similar to that of the α^Mg^γ heterodimer (PDB code 5GRH)^13^, with an RMSD of 0.5 Å. Both the α and γ subunits comprise of 10 α-helices (α1-α10) and 12 β-strands (β1–β12), which fold into a large domain, a small domain, and a clasp domain (Fig. 3a). The dimeric interface of the αγ heterodimer is primarily mediated by a number of conserved residues from the α6 and α7 helices of the small domain and the β6 and β7 strands of the clasp domain of both subunits via both hydrophilic and hydrophobic interactions.

### Structure of the active site

**Figure 4:**
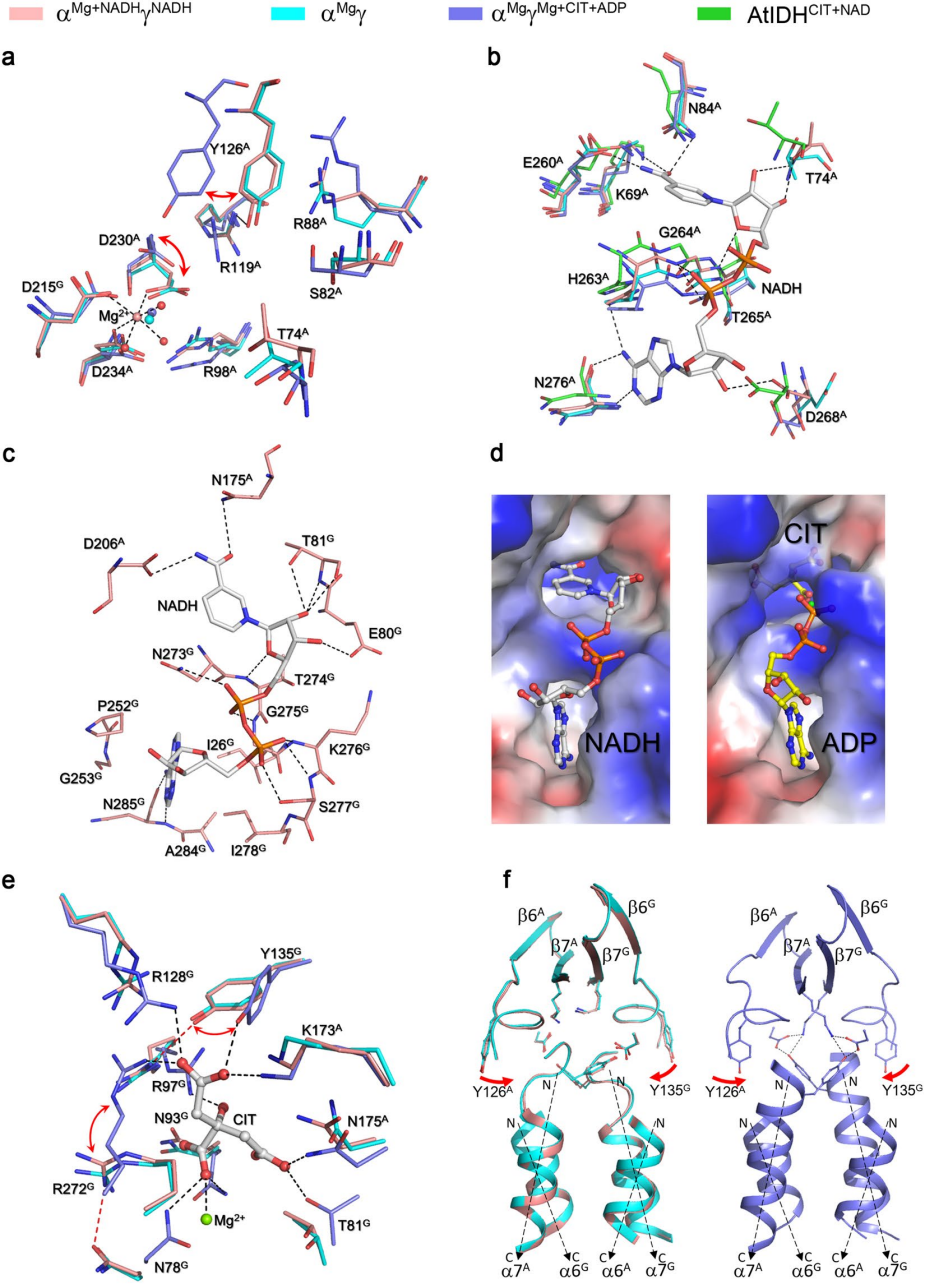
Structures of the active site and the allosteric site in the α^Mg+NADH^γ^NADH^ heterodimer. (**a**) Comparison of the ICT- and Mg^2+^-binding sites in the α^Mg+NADH^γ^NADH^ (salmon), α^Mg^γ (cyan, PDB code: 5GRH) and α^Mg^γ^Mg+CIT+ADP^ (slate, PDB code: 5GRE) structures. The residues composing the active site are shown in stick models, and the metal ion and water molecules as spheres. For clarity, only the coordination bonds of the metal ion in the α^Mg+NADH^γ^NADH^ structure are indicated with dashed lines. (**b**) Comparison of the NAD^+^/NADH-binding site at the active site in the α^Mg+NADH^γ^NADH^ (salmon), α^Mg^γ (cyan), α^Mg^γ^Mg+CIT+ADP^ (slate), and *A. thiooxidans* NAD-IDH (green, PDB code: 2D4V) structures. For clarity, only the NADH in the α^Mg+NADH^γ^NADH^ structure is shown in stick model, and its hydrogen-bonding interactions with the surrounding residues are indicated with dashed lines. (**c**) Structure of the allosteric site in the α^Mg+NADH^γ^NADH^ structure. The NADH is shown in stick model, and its hydrogen-bonding interactions with the surrounding residues are indicated with dashed lines. (**d**) Comparison of the allosteric site in the α^Mg+NADH^γ^NADH^ (left) and α^Mg^γ^Mg+CIT+ADP^ (right) structures. The protein is shown with electrostatic potential surface, the bound NADH, CIT and ADP are shown with ball-and-stick models, and the Mg^2+^ with a green sphere. The NADH occupies a large portion of the binding sites for CIT, Mg^2+^ and ADP in the γ subunit. (**e**) Comparison of the allosteric site in the α^Mg+NADH^γ^NADH^ (salmon), α^Mg^γ (cyan), and α^Mg^γ^Mg+CIT+ADP^ (slate) structures. The bound CIT in the α^Mg^γ^Mg+CIT+ADP^ structure is shown in stick model and colored in gray, the metal ion is shown as a green sphere, and the hydrogen-bonding interactions between CIT and the surrounding residues are indicated with black dashed lines. The hydrogen-bonding interactions between Arg97^G^-Tyr135^G^ and between Asn78^G^-Arg272^G^ in the α^Mg+NADH^γ^NADH^ structure are indicated with red dashed lines. (**f**) Comparison of the heterodimer interface in the α^Mg+NADH^γ^NADH^ (salmon), α^Mg^γ (cyan), and α^Mg^γ^Mg+CIT+ADP^ (slate) structures. Left: the heterodimer interface in the α^Mg+NADH^γ^NADH^ (salmon) and α^Mg^γ (cyan) structures. Right: the heterodimer interface in the α^Mg^γ^Mg+CIT+ADP^ (slate) structure. The hydrogen-bonding interactions between the N-terminal of the α7^A^ and α7^G^ helices and the β7^A^ and β7^G^ strands in the α^Mg^γ^Mg+CIT+ADP^ structure are indicated with dashed lines.

The active site resides in the deep cleft formed largely by the small and large domains of the α subunit and the small domain of the γ subunit, which consists of the binding sites for ICT, Mg^2+^, and NAD^+^ adjacently (Fig. 3a). Previous biochemical and structural studies have shown that in the αγ heterodimer, Asp230^A^, Asp234^A^ and Asp215^G^ (superscripts “A” and “G” denote the α subunit and γ subunit, respectively) form the Mg^2+^-binding site, and Thr74^A^, Ser82^A^, Arg88^A^, Arg98^A^, Arg119^A^, Tyr126^A^ and Asp230^A^ form the ICT-binding site^8,9,11,13^. The residues of the α subunit are unvaried in the corresponding subunits of all NAD-IDHs (Fig. 3c). In the α^Mg+NADH^γ^NADH^ heterodimer, the key residues composing the ICT- and Mg^2+^-binding sites and particularly the side chains of Tyr126^A^ and Asp230^A^ exhibit similar conformations as those in the inactive α^Mg^γ heterodimer (PDB code 5GRH) but different conformations from those in the active α^Mg^γ^Mg+CIT+ADP^ heterodimer (PDB code 5GRE)^13^ (Fig. 4a). Specifically, like in the α^Mg^γ structure, Tyr126^A^ forms a hydrogen bond via its side chain with the side chain of Arg119^A^ and a cation-π interaction with the side chain of Arg88^A^, and Asp230^A^ forms a coordination bond via its side chain with the Mg^2+^. In contrast, in the α^Mg^γ^Mg+CIT+ADP^ structure, the side chain of Tyr126^A^ is oriented towards the ICT-binding site and the side chain of Asp230^A^ is oriented towards the side chain of Tyr126^A^, and consequently the side chains of these two residues forms a hydrogen bond and additionally both of the side chain and main chain of Asp230^A^ make a coordination bond with the Mg^2+^. These results indicate that the active site in the α^Mg+NADH^γ^NADH^ heterodimer assumes an inactive conformation as that in the α^Mg^γ heterodimer, corresponding to the enzymatic state with a high *S*_0.5,ICT_ (the concentration of the substrate ICT needed to 0.5*V_max,ICT_, where “*V*_max,ICT_” is the maximal velocity in the presence of saturated ICT)^12,13^.

In the α^Mg+NADH^γ^NADH^ structure, the NAD^+^-binding site is bound with an NADH: the adenine moiety of the NADH is located at the deep pocket of the active site and the nicotinamide moiety positioned in close to the ICT-binding site. The NADH makes hydrogen-bonding interactions with a number of residues from the large domain of the α subunit, including Lys69^A^, Thr74^A^, Asn84^A^, Glu260^A^, His263^A^, Gly264^A^, Thr265^A^, Asp268^A^, and Asn276^A^ (Fig. 4b). Specifically, the nicotinamide moiety of NADH makes hydrogen-bonding interactions with the side chains of Lys69^A^, Asn84^A^ and Glu260^A^; the nicotinamide ribose moiety forms hydrogen bonds with the main chains of Thr74^A^ and Thr265^A^; the phosphate and the ribose 2′-OH of the adenosine moiety form a hydrogen bond with the main chain of Gly264^A^ and the side chain of Asp268^A^, respectively; and the adenine moiety forms a hydrogen bond with the side chain of His263^A^ and two hydrogen bonds with the main chain of Asn276^A^. Sequence alignment shows that the residues involved in the NADH binding are also strictly conserved in the catalytic subunits of all NAD-IDHs (Fig. 3c). Structural comparison also shows that these residues assume very similar conformations as those in both the inactive α^Mg^γ heterodimer and the active α^Mg^γ^Mg+CIT+ADP^ heterodimer^13^, indicating that in the absence of ICT, the active site of the αγ heterodimer can bind NAD^+^/NADH, and the NAD^+^/NADH binding induces no notable conformational changes at the active site. These results concur with the previous biochemical data showing that NAD-IDH adopts an ordered mechanism with the NAD^+^ binding prior to the ICT binding^3^, and our biochemical data showing that NADH can compete with NAD^+^ to bind at the active site and inhibits the activity of the αγ heterodimer (Figs 1 and 2, and Table 1).

Comparison with the structures of *Acidithiobacillus thiooxidans* NAD-IDH and NADP-IDHs also allows us to identify the residues involved in the recognition of NAD^+^ and NADP^+^, which differ only between the 2′-hydroxyl group of the adenosine ribose of NAD^+^ and the 2′-phosphomonoester group of the adenosine ribose of NADP^+^. In the α^Mg+NADH^γ^NADH^ structure, Asp268^A^ makes a hydrogen-bonding interaction with the 2′-hydroxyl group of the adenosine ribose, and in the *A. thiooxidans* NAD-IDH structure (PDB code 2D4V), the equivalent residue Asp357 forms two hydrogen-bonding interactions with the 2′- and 3′- hydroxyl groups of the adenosine ribose of NAD^+^ ^15^. Sequence alignment shows that this Asp is invariable in the corresponding subunits of human, *Xenopus laevis*, and *Danio rerio* NAD-IDHs (Fig. 3c). However, in the NADP-bound human cytosolic NADP-IDH structure (PDB code 1T0L), Arg314 and His315 make hydrogen-bonding interactions with the 2′-phosphomonoester group of the adenosine ribose of NADP^16^ and the equivalent residues are also unvaried in all the dimeric eukaryotic NADP-IDHs^17^. These results indicate that Asp268^A^ of human NAD-IDH (or the equivalent of other NAD-IDHs) is the specificity determinant for NAD^+^ against NADP^+^, and Arg314 and His315 of human cytosolic NADP-IDH (or the equivalents of other NADP-IDHs) are the specificity determinants for NADP^+^ against NAD^+^.

### Structure of the allosteric site

The allosteric site resides in the deep cleft formed largely by the small and large domains of the γ subunit as well as the small domain of the α subunit, which comprises the binding sites for CIT, Mg^2+^, and ADP adjacently (Fig. 3a). Surprisingly, in the α^Mg+NADH^γ^NADH^ structure, there is an NADH bound at the allosteric site, which occupies a large portion of the allosteric site. Compared with the α^Mg^γ^Mg+CIT+ADP^ structure^13^, the ADP moiety and nicotinamide-ribose moiety of NADH occupy fully the ADP-binding site and the nicotinamide moiety of NADH occupies the Mg^2+^-binding site and part of the CIT-binding site, making extensive hydrophilic and hydrophobic interactions with the surrounding residues (Fig. 4c,d). Specifically, the adenine moiety forms numerous hydrophobic interactions with Ile26^G^, Pro252^G^, Gly253^G^, Ile278^G^ and Ala284^G^, and additionally two hydrogen bonds with the main chain of Asn285^G^; the adenosine phosphate forms two hydrogen bonds with the main chain and side chain of Ser277^G^ and a hydrogen bond with the main chain of Lys276^G^; the nicotinamide phosphate forms one hydrogen bond each with the main chain of Gly275^G^ and the side chain of Asn273^G^; the nicotinamide ribose moiety forms three hydrogen bonds with the main chain and side chain of Thr81^G^, and additionally one hydrogen bond each with the side chain of Glu80^G^ and the main chain of Thr274^G^; and the nicotinamide moiety forms one hydrogen bond each with the side chain of Asn175^A^ and the side chain of Asp206^A^.

Intriguingly, detailed structural comparisons show that the residues making direct interactions with NADH at the allosteric site adopt very similar conformations to those in the α^Mg^γ structure instead of those in the α^Mg^γ^Mg+CIT+ADP^ structure^13^ (Fig. 4e,f). In other words, the CIT-binding induced conformational changes at the allosteric site and the heterodimer interface are not observed in the α^Mg+NADH^γ^NADH^ structure. Particularly, in the α^Mg+NADH^γ^NADH^ structure, the hydrogen-bonding interactions between the side chains of Arg97^G^ and Tyr135^G^ and between the side chains of Asn78^G^ and Arg272^G^ at the allosteric site are retained, and thus all the side chains of these residues are oriented outwards from the CIT-binding site. In addition, the N-terminal regions of the α7^A^ and α7^G^ helices at the heterodimer interface assume loop conformation instead of helical conformation, and make no hydrogen-bonding interactions with the β7^A^ and β7^G^ strands of the clasp β-sheet. These results indicate that NADH can bind to the allosteric site, which blocks the binding of the activators ADP and CIT, and the NADH binding induces no notable conformational changes at the allosteric site and the heterodimer interface and hence cannot activate the enzyme.

### Functional role of the NADH binding

**Figure 5:**
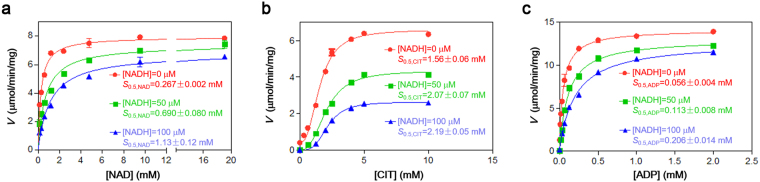
Effects of NADH on the activity and the CIT or ADP activation of the αγ heterodimer. (**a**) The NAD^+^ saturation curve in the absence and presence of NADH. The kinetic parameters for NAD^+^ were measured at the standard conditions with varied concentrations of NAD^+^ (0–20 mM) as described in “Methods”. The *V*_max,NAD_ in the presence of 0, 50, and 100 μM NADH are determined to be 7.95 ± 0.13, 7.38 ± 0.20, and 6.79 ± 0.21 μmol/min/mg, respectively. The *S*_0.5,NAD_ values are shown in the figure. (**b**) The activation curve of CIT in the absence and presence of NADH. The CIT activation curves were measured at subsaturating substrate (ICT) conditions with varied concentrations of CIT as described in “Methods”. The *V*_max,CIT_ in the presence of 0, 50, and 100 μM NADH are determined to be 6.58 ± 0.14, 4.34 ± 0.11, and 2.63 ± 0.05 μmol/min/mg, respectively. The *S*_0.5,CIT_ values are shown in the figure. (**c**) The activation curve of ADP in the absence and presence of NADH. The ADP activation curves were measured at subsaturating substrate (ICT) conditions with varied concentrations of ADP in the presence of 1 mM CIT as described in “Methods”. The *V*_max,ADP_ in the presence of 0, 50, and 100 μM NADH are determined to be 14.1 ± 0.2, 13.0 ± 0.1, and 12.7 ± 0.2 μmol/min/mg, respectively. The *S*_0.5,ADP_ values are shown in the figure.

Our biochemical data show that the αγ heterodimer contains at least two binding sites for NADH (Table 1). Consistently, our structural data confirm that the αγ heterodimer has two NADH-binding sites with one at the active site and the other at the allosteric site (Fig. 3). Previous biochemical studies suggested that the inhibitory effect of NADH is through its binding to the active site, which impedes the NAD^+^ binding and thus inhibits the enzymatic activity^5^. Indeed, our biochemical data show that NADH has an evident inhibitory effect on the activity of the αγ heterodimer with an IC_50_ of 82.7 ± 6.0 μM at the standard conditions (Fig. 1). Furthermore, in the presence of 50 μM and 100 μM NADH (corresponding to 30% and 60% inhibition of the activity), the *S*_0.5,NAD_ of the enzyme (the concentration of NAD^+^ needed to reach 0.5*V_max,NAD_, where “*V*_max,NAD_” is the maximal velocity in the presence of saturated NAD^+^) is substantially increased from 0.267 ± 0.002 mM to 0.690 ± 0.080 mM (by 2.6 folds) and 1.13 ± 0.12 mM (by 4.2 folds), respectively; however, the *V*_max,NAD_ at the saturation concentration of NAD^+^ is only slightly decreased (Fig. 5a). These results indicate that the NADH binding competes with the NAD^+^ binding, which is concurrent with the structural data showing that NADH can bind to the NAD^+^-binding site, and in the absence of the CIT and ADP activation, the inhibitory effect of NADH can be almost completely reversed by high concentration of NAD^+^. To investigate the functional role of NADH binding at the allosteric site, we examined the effect of NADH binding on the activation of CIT at the subsaturating substrate (ICT) conditions with varied concentrations of CIT (Fig. 5b). In the absence of NADH, the αγ heterodimer has an *S*_0.5,CIT_ of 1.56 ± 0.06 mM [the CIT concentration needed to reach 0.5*(*V*_max,CIT_-*V*_0,CIT_), where “*V*_max,CIT_” is the maximal velocity in the presence of saturated CIT concentration and “*V*_0_” is the initial velocity in the absence of CIT] and CIT has an evident activation effect on the enzyme with a *V*_max,CIT_ of 6.58 ± 0.14 μmol/min/mg. In the presence of 50 μM and 100 μM NADH, the *S*_0.5,CIT_ of the enzyme is slightly increased to 2.07 ± 0.07 mM and 2.19 ± 0.05 mM, but the *V*_max,CIT_ at the saturated CIT concentration is markedly decreased to about 66% and 40% of that in the absence of NADH, respectively. These results indicate that the NADH binding slightly decreases the binding of CIT at the allosteric site and thus increases the CIT concentration for activation, but the activation of CIT alone cannot fully reverse the inhibitory effect of NADH, which could be attributed to the binding of NADH to the active site. As the ADP activation is dependent on and synergistic with the binding of CIT^6,12,13^, we examined the effect of NADH on the ADP activation in the presence of 1 mM CIT (Fig. 5c). As expected, in the absence of NADH, the activity of the αγ heterodimer is markedly increased by addition of ADP and CIT, and the enzyme exhibits an *S*_0.5,ADP_ of 0.056 ± 0.004 mM [the ADP concentration needed to reach 0.5*(*V*_max,ADP_−*V*_0,ADP_), where “*V*_max,ADP_” is the maximal velocity in the presence of saturated ADP concentration and “*V*_0_” is the initial velocity in the absence of ADP] and a *V*_max,ADP_ of 14.1 ± 0.2 μmol/min/mg. In the presence of 50 and 100 μM NADH, the *S*_0.5,ADP_ is increased to 0.113 ± 0.008 mM (by 2.0 folds) and 0.206 ± 0.014 mM (by 3.7 folds), respectively, whereas the *V*_max,ADP_ is only slightly decreased to 13.0 ± 0.1 μmol/min/mg and 12.7 ± 0.2 μmol/min/mg, respectively. Moreover, the inhibitory effect of NADH is almost completely reversed at the saturation concentration of ADP and 1 mM CIT. These results indicate that the binding of NADH to the allosteric site also decreases the binding of ADP and increases the ADP concentration for activation. These data together demonstrate that NADH can compete with the binding of CIT and ADP at the allosteric site and thus impedes the activation effect of CIT and ADP, and the inhibitory effect of NADH can be almost completely reversed by high concentrations of ADP and CIT.

### Molecular mechanism of the inhibition of the αγ heterodimer by NADH

The previous biochemical studies have shown that the activity of the purified bovine heart NAD-IDH can be inhibited by NADH^5,14^; however, the molecular mechanism is unclear. It was suggested that the inhibitory effect of NADH is through its binding to the active site, which impedes the binding of NAD^+^ and thus impairs the enzymatic activity^5^. In this work, we demonstrate that the αβ, αγ and α_2_βγ enzymes of human NAD-IDH can also be inhibited by NADH and that all three enzymes have much higher binding affinity for NADH than NAD^+^, and the αβ heterodimer has the highest binding affinity for both NADH and NAD^+^ followed by the α_2_βγ heterotetramer and then the αγ heterodimer. We further solved the structure of the αγ heterodimer bound with an Mg^2+^ and an NADH at the active site and an NADH at the allosteric site and performed the kinetic studies. The structural and biochemical data together reveal the inhibitory mechanisms of NADH on the αγ heterodimer.

Our structural data show that NADH can bind to the NAD-binding site, which competes with the binding of the cofactor NAD^+^ at the active site. Consistently, our biochemical data show that the NADH binding markedly increases the *S*_0.5,NAD_ of the enzyme, and hence inhibits the activity of the enzyme. In addition, our structural data show that NADH can also bind to the allosteric site to occupy a large portion of the binding sites for the activators CIT and ADP, and this binding competes with the binding of CIT and ADP and thus impedes the activation of the enzyme. Indeed, our biochemical data show that the NADH binding increases the concentrations of the activators for activation, and hence inhibits the activity of the enzyme. These two effects together contribute to the inhibition of NADH on the enzymatic activity of the αγ heterodimer. This mechanism is very likely applicable to the α_2_βγ holoenzyme of human and other mammalian NAD-IDHs. Nonetheless, we cannot rule out the possibility that the active site and/or allosteric site in the α_2_βγ heterotetramer might have different conformations from these in the αγ heterodimer and thus the ligands might have different binding modes. Further structural and functional studies of the α_2_βγ heterotetramer will resolve this issue and provide a complete understanding of the molecular basis for the assembly and the allosteric regulation mechanism of the α_2_βγ heterotetramer.

## Methods

### Cloning, expression, and purification

Preparations of the αβ and αγ heterodimers and the α_2_βγ heterotetramer of human NAD-IDH followed the method described previously^13^. In particular, to obtain the αγ heterodimer, the human DNA fragments corresponding to the α and γ subunits of human NAD-IDH were amplified by PCR and cloned into the pQlinkN co-expression plasmid following the standard procedure^18^. For purification purpose, the C-terminal end of the γ subunit was attached with a TEV protease cleavage site followed by a His_6_ tag. The constructed pQlinkN-α-γ-tev-His_6_ plasmid was transformed into *E. coli* BL21(DE3) Codon-Plus strain (Novagen), and the bacterial cells were cultured in LB medium supplemented with 100 μg/ml ampicillin at 37 °C until OD600 reached 0.5. The protein expression was induced by adding 0.4 mM isopropyl-β-D-thiogalactopyranoside (IPTG) at 23 °C for 20 hr. The bacterial cells were harvested by centrifugation and resuspended in a lysis buffer [50 mM HEPES (pH 7.4), 200 mM NaCl, 0.2 mM MnCl_2_, 10% (w/v) glycerol, 7.2 mM β-ME, and 1 mM PMSF], and then lysed by sonication on ice. Protein purification was first carried out by affinity chromatography using an Ni-NTA column (Qiagen). The C-terminal His_6_-tag of the γ subunit was cleaved with the TEV protease by dialysis overnight against the lysis buffer. The protein mixture was reloaded on an Ni-NTA column and washed with the lysis buffer containing 10 mM imidazole, and the eluent was further purified by gel filtration using a Superdex 200 10/300 GL column (GE Healthcare). The purified protein was of high purity (above 95%) as analyzed by SDS-PAGE (12%), and stored in the storage buffer (10 mM HEPES, pH 7.4, 200 mM NaCl, and 5 mM β-ME) for the structural and biochemical studies.

### Crystallization, diffraction data collection and structure determination

To obtain the αγ heterodimer bound with NADH, the protein solution (10 mg/ml) was incubated with NADH (final concentration of 10 mM) at 4 °C before crystallization. Crystallization screening was carried out using the hanging drop vapor diffusion method, and crystals of the α^Mg+NADH^γ^NADH^ heterodimer were grown at 20 °C from drops consisting of 1 μl each of the protein solution and the reservoir solution [0.1 M HEPES (pH 7.5), 50 mM MgCl_2_, and 30% (v/v) PEGMME 550]. Diffraction data were collected from a flashed-cooled crystal at 100 K at beamline 19U1 of NFPSS, China, and processed, integrated and scaled together using HKL3000^19^. The flashed-cooled crystal was prepared by soaking the crystal into the cryo-protectant containing the reservoir solution mixed with 20% ethylene glycol and then quickly dipping into liquid N_2_. Crystallographic statistics are listed in Table 2.

The α^Mg+NADH^γ^NADH^ structure was solved with the molecular replacement (MR) method as implemented in program Phaser^20^ using the α^Mg^γ structure (PDB code 5GRH)^13^ as the search model. Structure refinement was performed using programs Phenix^21^ and REFMAC5^22^, and manual model building with program Coot^23^. Structural analyses were performed using programs in the CCP4 suite^24^ and the PISA server^25^. The structure figures were generated using PyMol^26^ and the structure-based sequence alignment figure using ESPpript 3.0^27^. Statistics of the structure refinement and the quality of the structure model are also listed in Table 2.

### Enzymatic activity assays and kinetic analyses

The enzymatic activity assay was carried out using a method as described previously^13^. The activity of the enzyme was determined by monitoring the reduction of NAD^+^ to NADH over time at the specific absorption wavelength of NADH (340 nm) using a Beckman Coulter DU 800 spectrophotometer. The standard reaction solution consisted of 33 mM Tris-acetate (pH 7.4), 20 mM _DL_-ICT, 2 mM Mn^2+^, and 1 mM NAD^+^ in a total volume of 1 ml. The catalytic reaction was initiated by addition of NAD^+^. The production of NADH follows pseudo-first-order kinetics and the time course of the product appearance is well modeled by a linear function up to the time when about 10% of the total substrate was converted to the product. The initial velocity (*V*) was determined from the slope of a linear fit of the early time point data. The activity of the enzyme is defined as the velocity at the specific conditions, which is expressed as μmol of NADH produced per min per mg of enzyme (μmol/min/mg). All experiments were carried out at 25 °C and repeated at least twice under the same conditions.

The activities of the αγ and α_2_βγ enzymes in the presence of NADH were determined at the standard conditions with varied NADH concentrations, and the activity of the αβ enzyme was determined at the same conditions with a higher concentration of MnCl_2_ (50 mM). The inhibitory effect of NADH on the activity of the enzyme is presented as percent of inhibition (I) as a function of NADH concentration. I = (*V*_0_ − *V*)/*V*_0_ * 100, where *V*_0_ and *V* were the initial velocity in the absence and presence of NADH, respectively.

To analyze the effect of NADH on the binding of the cofactor NAD^+^, the apparent kinetic parameters of the enzyme for NAD^+^ in the presence of 0, 50 and 100 μM NADH were determined at the standard conditions with varied NAD^+^ concentrations (0–20 mM). The values of the *V*_max,NAD_ and the *S*_0.5,NAD_ were determined by fitting the experimental data into the Michaelis-Menten equation “*V* = *V*_max_ * [S]/(*S*_0.5_ + [S])” using program Graphpad Prism (Graphpad Software), where “*S*_0.5_” is the NAD^+^ concentration at the velocity of 0.5 * *V*_max_, and “[S]” is the concentration of NAD^+^.

To analyze the effects of NADH on the binding of the activators CIT and ADP, the activation curve for CIT in the presence of 0, 50, and 100 μM NADH were determined at subsaturating substrate (ICT) conditions (33 mM Tris-acetate, pH 7.4, 0.6 mM _DL_-ICT, 2 mM Mn^2+^, and 3 mM NAD^+^) with varied CIT concentrations (0–10 mM), and the activation curve for ADP in the presence of 0, 50, and 100 μM NADH were determined at the same conditions with varied ADP concentrations (0–2 mM) and the presence of 1 mM CIT. The apparent kinetic parameters for CIT (or ADP) were obtained by fitting the experimental data into the non-Michaelis-Menten equation “*V* *−* *V*_0_ = (*V*_max_ − *V*_0_) * [S]^h/(*S*_0.5_^h + [S]^h)”, where “h” is the Hill coefficient, “*S*_0.5_” is the CIT (or ADP) concentration when (*V* − *V*_0_) equals to 0.5 * (*V*_max_ − *V*_0_), “[S]” is the concentration of CIT (or ADP), “*V*_0_” is the initial velocity in the absence of CIT (or ADP), and “*V*_max_” is the maximal velocity in the presence of saturated CIT (or ADP) concentration. “*V*_max_−*V*_0_” reflects the maximal activation effect caused by addition of CIT (or ADP). The h value for CIT activation in the presence of 0, 50, and 100 μM NADH is about 2.5, 2.6, and 3.4, respectively, indicating the CIT binding exhibits significant cooperativity. The h value for ADP activation in the presence of 0, 50, and 100 μM NADH is all about 1, indicating that the ADP binding in the presence of 1 mM CIT exhibits no cooperativity. All of the values are the averages of three independent experiments with standard errors.

### Isothermal titration calorimetry analyses

Isothermal titration calorimetry (ITC) measurements were performed at 20 °C using an ITC200 Micro-Calorimeter Q6 (MicroCal). An initial injection of 0.4 μl titrant (NADH or NAD^+^) was discarded for each dataset to remove the effect of titrant diffusion across the syringe tip during the equilibration process. The αβ, αγ or α_2_βγ protein (30–200 μM) (titrand) was first dialyzed against the buffer (50 mM HEPES, pH 7.4, and 200 mM NaCl) and then placed in the sample cell with a reaction volume of 200 μl. The titrant (0.8–8 mM) was prepared in the same buffer as the titrand, and aliquots of the titrant (2 μl each) were injected incrementally into the sample cell at an interval of 120 sec (or 130 sec or 150 sec) with constant stirring at 750 rpm. The exact concentrations of titrand and titrant used in the measurements are listed in Table 1. For each concentration of titrant, a background titration was performed using identical titrant with the buffer solution placed in the sample cell to obtain the heat of dilution, which was subtracted from the measured heat change. The normalized corrected heat change of injectant was plotted against the molar ratio of titrant vs. titrand. The values of thermodynamic parameters were derived from the titration curve by fitting the experimental data using a nonlinear least-squares method with the single set of binding sites model implemented in MicroCal Origin software version 7.0. The dissociation constant (*K*_d_) equals to reciprocal of the association constant (*K*_a_).

### Protein Data Bank accession code

The α^Mg+NADH^γ^NADH^ structure has been deposited with the RCSB Protein Data Bank under accession code 5YVT.
